# Black Tea Samples Origin Discrimination Using Analytical Investigations of Secondary Metabolites, Antiradical Scavenging Activity and Chemometric Approach

**DOI:** 10.3390/molecules23030513

**Published:** 2018-02-26

**Authors:** Wojciech Koch, Wirginia Kukula-Koch, Łukasz Komsta

**Affiliations:** 1 Chair and Department of Food and Nutrition, Medical University of Lublin, 4a Chodźki Str., 20-093 Lublin, Poland; kochw@interia.pl; 2 Chair and Department of Pharmacognosy with Medical Plant Unit, Medical University of Lublin, 1 Chodźki Str., 20-093 Lublin, Poland; 3 Department of Medicinal Chemistry, Medical University of Lublin, 4 Jaczewskiego str., 20-090 Lublin, Poland; lukasz.komsta@umlub.pl

**Keywords:** *Camellia sinensis*, black teas, catechins, antioxidant activity, LC-ESI-Q-TOF-MS, principal component analysis

## Abstract

A comprehensive study on the composition and antioxidant properties of black tea samples with a chemometric approach was performed via LC-ESI-Q-TOF-MS, DPPH radical scavenging assay, and Folin–Ciocalteu assay (TPC). Marked differences between the teas from seven different countries (China, India, Iran, Japan, Kenya, Nepal, Sri Lanka) were shown. The Indian samples demonstrated the highest total catechin content (184.8 mg/100 mL), the largest TPC and DPPH scavenging potential (58.2 mg/100 mL and 84.5%, respectively). The applied principal component analysis (PCA) and ANOVA revealed several correlations between the level of catechins in tea infusions. EC (epicatechin), ECG (epicatechin gallate), EGC (epigallocatechin), and EGCG (epigallocatechin-3-gallate) content was not correlated with DPPH, gallic acid, and TPC; however, a strong correlation of EC and ECG between themselves and a negative correlation of these two catechins with EGCG and EGC was noted. Interestingly, simple catechins were not found to be responsible for antioxidant properties of the black teas. The samples collected in the higher altitudes were similar.

## 1. Introduction

Tea is the most widely consumed drink across the world after water [1]. Every day almost 2/3 of the world’s population drinks 18–20 billion cups of tea, and its annual production is estimated to be around 2.9 million tons [2]. Tea ((*Camellia sinensis* (L.) Kuntze)) belongs to the family Theaceae and is cultivated in over 45 countries across all continents except North America and Antarctica. Naturally, the best growth of tea is observed in tropical and subtropical climate with sufficient amount of precipitation on a slightly acidic soil [3]. Tea as a food product is obtained from young leaves, immature buds, and delicate stalks of tea bush, which are then processed. Based on the type of production process, *C. sinensis*-based teas can be divided into black, green, Pu-erh, oolong, yellow, and white teas [4,5]. However, recent research demonstrated that *C. sinensis* also can be divided into seven types, namely green, yellow, white, oolong, black, aged pu-erh and ripened pu-erh [6]. Green tea is produced from freshly harvested leaves that are immediately steamed to prevent enzymatic inactivation [7]. Furthermore, white tea is a non-fermented product obtained from first, young leaves and buds harvested in spring before their maturation. It is considered as the most high quality type of tea. Only black tea is fully fermented, obtained under the influence of warm air and oxidizing enzymes [8].

The production of black tea accounts for over 75% of the whole tea production in the world; therefore, it can be considered as the most important kind of tea for general population. Although green tea is considered as the most active and rich in phenolic compounds, recent studies have shown that black tea also possesses very high biological activity [9,10]. It contains basic polyphenolic compounds called as catechins, such as (–)-gallocatechin (GC), (–)-epicatechingallate (ECG), (–)-epigallocatechin (EGC), (+)-catechin (C), (–)-epicatechin (EC), (–)-gallocatechingallate (GCG), and (−)-epigallocatechin-3-gallate (EGCG) and also complicated polymers such as theaflavins (TFs), thearubigins (TRs), and theabrownins (TBs) [11,12]. The latter complex phenolic compounds (TFs, TRs, and TBs) emerge in the fermentation process as a result of oxidation of catechins and their gallates during production of black teas [12]. Although black tea infusions are considered to have lower antioxidant activity compared to green tea, they possess a wide spectrum of biological properties because of high concentration of simple catechins as well as TFs or TBs [13]. Black tea infusions are known to have antimutagenic and anticarcinogenic properties. They accelerate cell apoptosis and prevent the occurrence of some cancer types (lungs, skin, liver, or pancreas) [10,14,15]. Furthermore, they are characterized by strong antibacterial, antiviral, and antifungal properties [9,16,17]. Recent studies suggest that black teas decrease blood pressure and cholesterol level, especially in patients with high fat intake, which can be crucial in the prevention of cardiovascular diseases [18,19].

Even though various pharmacological activities of black tea samples have been studied so far, no publications can be found, which report the actual compositional differences between the components of black tea in relation to their origin.

Black tea leaf infusions contain a large number of compounds of natural origin as well as some obtained during fermentation process; therefore, chemometric tools can complement the chromatographic analysis. The application of principal component analysis (PCA) allows treating a sample of tea as a point in a multivariate space. Furthermore, some intercorrelations between all constituents can be examined and all variability of content can be presented as several independent (orthogonal) trends. If the constituents are intercorrelated, the whole dataset can be compressed to several trends, explaining almost all information inside the results.

The majority of the studies published deal with the analysis of green tea catechins with chemometric approach helping to elucidate chromatographic analysis [11,20]. Therefore, the primary goal of this study was to quantitatively differentiate catechins’ composition, which is scarcely published in the scientific literature, but also total phenolic content (TPC), and antioxidant activity of black tea infusions and classify them according to their origin. The secondary goal of this work was to find the correlations between the chemical composition of different origin tea samples using PCA-based methodology. To our knowledge, a study of this kind has not yet been performed so far, regarding the differences in catechin′s concentration in the infusions, antioxidant activity of black teas from different regions and correlations between these parameters. Because the tea is not consumed as a fresh product but only as a beverage, all results were expressed per 100 mL of the infusion.

## 2. Results and Discussion

### 2.1. LC-ESI-Q-TOF-MS Determination of Catechin Content

The identification of these phenolic compounds was obtained in a tailored chromatographic method applying a gradient of two solvents: acetonitrile and water, with an addition of formic acid, which increases conductivity in the ESI (electrospray ionization) source, but also suppresses ionization of phenolic compounds during this chromatographic step and finally improves the peak shape.

The operation parameters of the applied mass spectrometer enabled the ionization of the constituents of tea infusions and the MS/MS fragmentation spectra (see Supplementary Information File, Figure S1). The selected capillary voltage and the fragmentor voltage provided a successful analysis of these compounds. The collision-induced dissociation (CID) energy values set at 10 and 20 V induced a sufficient and not excessive ions’ fragmentation, which was more clear in case of the former value, because of a clearly visible deprotonated molecule in the spectrum. Negative ionization mode was found preferable for the analysis of these compounds, with the signal ca. 10 times more intensive than in the positive one.

All studied catechins were separated on the column with retention times: 4.8 min for GA, 10.9 min for EGC, 11.5 min for C, 12.3 min for EC, 12.8 for EGCG, 14.1 min for EGC. Based on that separation, we were able to do an analysis of the fragmentation patterns. The investigated gallates of catechins were found to lose the gallate group in the fragmentation process. Furthermore, the opening of the flavan-3-ol ring resulted in the presence of the following *m*/*z* value: 137 *m*/*z*.

The elaborated chromatographic method was checked for its suitability in the determination of GA and catechins. The linearity range for the studied compounds in the optimized method was within the range 0.03–12 µg/mL and the LOD and LOQ values were presented in the Materials and Methods chapter.

### 2.2. The Determination of Catechin Content in the Investigated Samples

Our study revealed the presence of four catechins (EGC, EGCG, ECG, and EC) and gallic acid (GA) in the tea infusions, which is in accordance with other investigations on tea catechins [6,21,22,23]. Moreover, Tao and co-workers suggest that EGCG, ECG, and EGC are the most abundant catechins in all three types of tea (green, black, and oolong), which together account for over 80% of all catechins [24]. Table 1 presents the average content of GA and catechins determined in the investigated black teas. 

Only for teas cultivated in Nepal, (both regions), all catechins were determined in the extracts, however, the total concentration of all catechins and GA in these teas was not high. The sum of all five compounds was the highest in Indian teas because of a very high concentration of EGC (178 mg/100 mL of tea infusion), which was also the main catechin in all investigated teas. This is in agreement with our recent study, which proved EGC to be the main simple catechin in black tea infusions [25]. The second group of teas rich in catechins were those cultivated in Sri Lanka with a total concentration of 54.1 mg/100 mL of infusion. However, the sum of all investigated catechins in the Iranian samples was calculated as 10.5 mg/100 mL only, with an average concentration of EGC of 9.91 mg/100 mL. It was the only group of samples in which EGCG was not detected. ECG and EC were the catechins present in the lowest concentrations in all tested teas (far below 1 mg/100 mL). Furthermore, ECG was detected only in the Iranian and Nepal samples. EC was not detected in teas from China, India, and Sri Lanka.

According to Khokhar and co-workers, EGCG and EGC are the predominant catechins in black and green teas. They also suggest that the levels of (+)-catechin are below the detection limit, which is probably due to a very low concentration of (+)-catechin in the tea leaves and a strong degradation of this compound during the fermentation process of black tea [21].

Still, the application of a high-resolution mass spectrometry (HR-MS) in this study—and namely a Q-TOF-MS apparatus—resulted in the identification of catechin in the obtained extracts, although its quantity was scarce (<0.1 µg/100 mL). Therefore, catechin was not quantified in the investigated samples. The above cited findings of Khokhar and co-investigators [21] are consistent with our results; however, our data suggest that the primary catechin in black tea is not EGCG but EGC. This may be connected with the hydrolysis of EGCG during storage and the production process of black teas. In all black teas investigated in this study, the concentration of EGC was much higher compared to EGCG. These data are in agreement with previous studies suggesting that EGCG is the primary catechin in green and oolong tea, but not in the black tea [24].

### 2.3. Antioxidant Activity Assessment and TPC in the Studied Samples

Not only green, but also black tea extracts possess strong antioxidant activity, which indicates that, not only simple catechins, but also TFs and TRs have high biological potential [23]. The review made by Wiseman and co-workers on antioxidants in tea suggests that green and black tea infusions have equally strong antioxidant activity [26]; however, according to Han and Chen, a comparison of relative inhibitory potency between simple catechins and TFs may be difficult to perform [27]. According to the previous findings, black tea infusions may be an important antioxidant-based food product in human nutrition.

Results of TPC and antiradical activity (Table 2) were in good agreement with chromatographic analysis of GA.

The teas cultivated in India were characterized by the highest phenolic content (expressed as GAE) and antioxidant activity. As it was previously described, these products contained the highest amounts of EGC, which was far above the concentrations determined in other investigated teas. High concentration of phenolics and high antiradical activity was also determined for teas from Nepal and Kenya. However, samples from China, Japan, and Iran used in this study were characterized by low antioxidant activity, which was well correlated with TPC and catechin content, which also were significantly lower compared to other teas. It should be strongly emphasized that not only simple catechins, but also condensed compounds such as TRs, TFs, and TBs, possessed a wide spectrum of biological properties, including antioxidant activity. Therefore, in this study, the Folin–Ciocalteu method was used, as a quick and simple tool for fast characterization of TPC content in black tea. Although this method is not specific, it could extend chromatographic data, especially for products with complex polyphenolic profile as black teas.

### 2.4. Chemometric Analysis of the Obtained Results

To investigate the dependences between determined compounds and sample properties, a scaled PCA was applied to the whole data matrix consisting of rows expressing the samples’ type and columns containing their properties. The following seven variables were used: GA, EGC, EGCG, ECG, and EC content (all expressed in mg/100 mL), DPPH (%) and TPC (mg/100 mL). 

**Figure 1:**
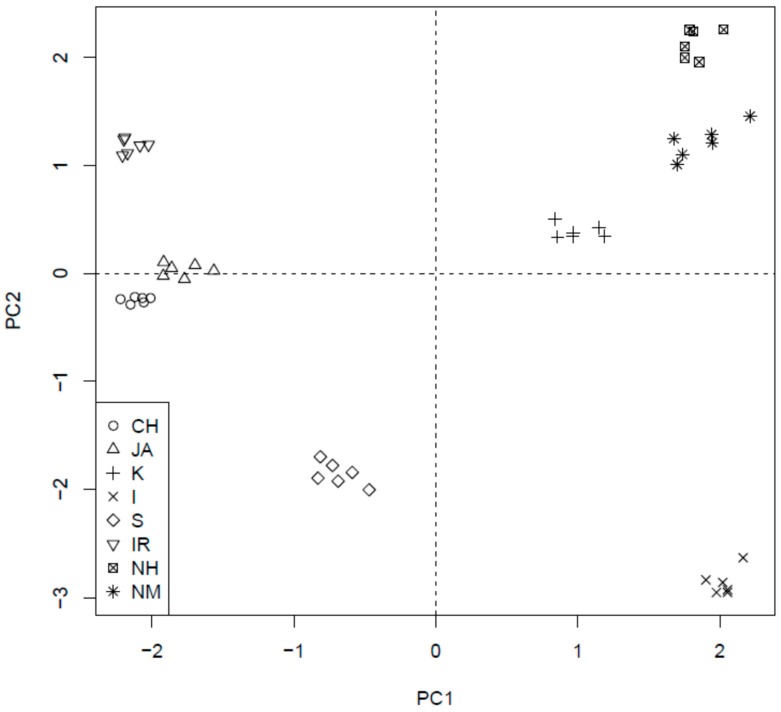
The scores of first two principal components of PCA analysis, explaining together 80% of information in the obtained dataset.

**Figure 2:**
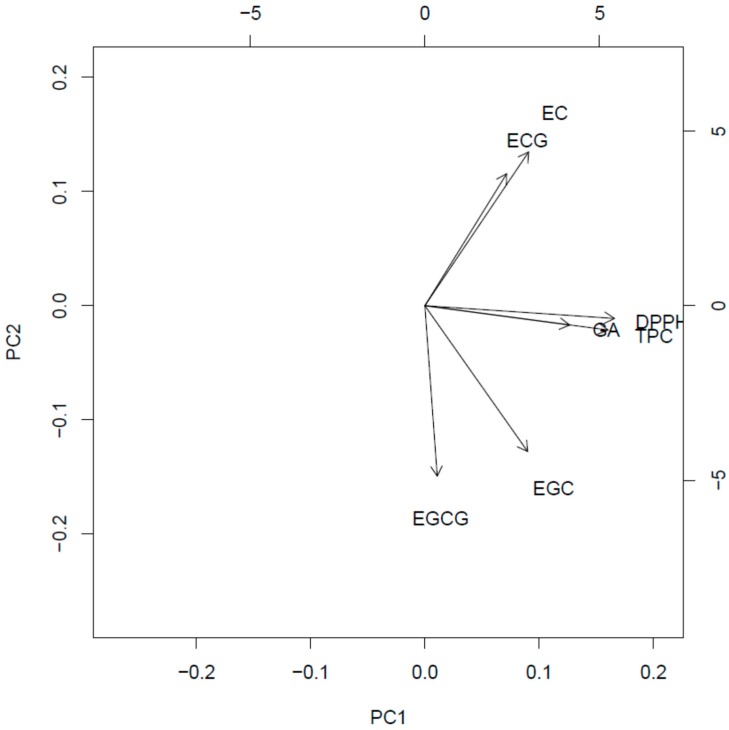
The loadings of first two components depicted on Figure 2. For explanations, see text.

Figure 1 presents the resulted scores and their corresponding loadings are placed in Figure 2.

The first principal component (PC1) explained the 45.2% of the total variance, connected with antioxidant activity. GA, TPC, and DPPH are strictly correlated with themselves; therefore, samples lying on the right side of score plot are characterized by the highest antioxidant activity (I, NM, and NH). The samples located on the left side (low PC1 value) are characterized by lowest antioxidant activity (CH, JA, and IR).

The second principal component (PC2) explains 35.3% of the samples’ variance. It represents the (independent to PC1) trend, connected with changes in EC, ECG, EGC, and EGCG content. The loading vectors of these compounds are located along the vertical axis. It means that they are almost uncorrelated with DPPH, GA, and TPC. EC and ECG are strictly correlated between themselves and they are visibly negatively correlated with EGCG and EGC, which form the second intercorrelated group. Therefore, a high PC2 value means high content of ECG and EC with simultaneous low content of EGCG and EGC (for example IR, NM, and NH). The low PC2 value indicates an opposite behavior (high EGCG and EGC with low ECG and EC, for example I and S). 

There is a high variability of the samples in the context of both of these trends and one can find samples representing all possible combinations of low/high PC1 and PC2 values. This fact results in the conclusion that catechins are almost not connected with antioxidant activity, whereas there is a visible correlation between their content in the investigated samples (including negative between two groups). Performed ANOVA corroborated significant differences between investigated parameters. This study confirmed that, because of the low catechin concentration in the black tea infusions (in comparison to the green tea), other compounds of phenolic nature are predominantly responsible for strong antioxidant properties of the black tea. For details, see Figures S2–S3 and Tables S1–S4 in the Supplementary Material.

The applied chemometric approach did not reveal any correlation between catechins’ content, antioxidant activity, and geographical origin. Fraser and co-workers [28,29] reported potential use of secondary metabolites analysis using different chemometric tools in the origin discrimination of black teas. However, their results were based on different parameters and revealed significant differences between the tea type (black, oolong and green), rather than location. In the present study, an interesting trend was also observed—the teas cultivated in the countries or regions located on the higher altitude above the sea level (Nepal and Kenya) were characterized by different—similar to one another—catechin content and antiradical properties in comparison to other tested samples (see Figure 2). There are several studies on different groups of secondary metabolites, which shed light on the relationship between the content of natural products in the extracts and the altitude of the cultivars’ occurrence [30,31]. Some of them have analyzed the levels of catechins in tea samples, but the conclusions drawn were inconclusive. Han and co-investigators [31] suggested that higher altitudes along with related lower temperatures induced the composition of Chinese green tea samples, leading to a better tea quality (based on the composition). However, Chen and co-workers [30], who investigated oolong tea samples, drew a conclusion that both the level of galloylation and the catechins’ concentration were inversely correlated with the altitude of the cultivar.

Our studies preliminarily suggest the presence of a correlation between the parameters described above also in black tea samples.

## 3. Materials and Methods

### 3.1. Plant Material

The investigated tea samples were purchased in Poland in 2015–2016 from professional tea shops. In total, 48 black teas, cultivated in seven countries, were used in this study. For each tea origin, a special code was given, which was presented in Table 3. The origin of the teas was guaranteed be the seller, which specializes in the trade of high quality, premium teas. No blended products were used in this study. In general, 864 black tea samples were investigated (8 geographic regions × 6 representatives × 6 batch numbers purchased × 3 samples taken). Table 3 shows a summary of the information on the number of replicate tea samples from each sampling region.

### 3.2. Reagents

Sodium carbonate (Na_2_CO_3_, reagent grade), citric acid and Folin–Ciocalteu reagent were obtained from Stanlab (Lublin, Poland). The solvents for liquid chromatography coupled with mass spectrometry (LC-MS) (spectrophotometric grade) such as acetonitrile, water, and formic acid were purchased from J.T. Baker (Center Valley, PA, USA). The standards of all investigated catechins (EGC, EGCG, ECG, and EC), trolox, gallic acid and 2,2-diphenyl-1-picrylhydrazyl (DPPH) radical were purchased from Sigma-Aldrich (St. Louis, MO, USA). All other reagents used for extraction were of reagent grade.

### 3.3. Preparation of Tea Infusions

Each tea was first milled using an electrical mill (type WZ-1, ZBPP, Poland). To prepare aqueous extracts, according to the conventional tea brewing method [32], about 1.0 g (±0.001 g) tea was weighed and transferred to a 250 mL conical flask. Then, brew was prepared using 100 mL of boiling distilled water and infused for 5 min under the watch glass cover. Because a moderate acid environment of the tea solution plays a pivotal role in the stability of the aqueous tea sample, after the extraction, pH of the extract was adjusted to 3.2 using citric acid, and it was diluted five times with distilled water (only for chromatographic determinations) [21,33]. Subsequently, the solution was filtered through 0.45 µm filter (Cronus, Gloucester, UK) prior to LC-MS analysis.

### 3.4. LC-MS Analysis

The LC-MS system consisted of an electrospray ionization with quadrupole time-of-flight mass spectrometer (ESI-Q-TOF-MS 6500 Series mass spectrometer, Agilent Technologies, Santa Clara, CA, USA) and an LC system (1200 Series, Agilent Technologies) composed of an autosampler, a degasser, a DAD detector, and a binary pump. The analytes were separated on a Zorbax RP 18 column from Agilent Technologies (size 150 mm × 2.1 mm, dp = 3.5 µm) at a flow rate of 0.2 mL min^−1^ and temperature of 20 °C. The mobile phase consisted of a combination of solvent A (0.1% formic acid in water) and solvent B (acetonitrile). The gradient elution was as follows: t = 0 min, 10% B; t = 10 min, 40% B; t = 12 min, 40% B; t = 17 min, 95% B; t = 20 min, 10% B; t = 22 min, 10% B and the stop time was set at 30 min. The photodiode array detector continuously recorded the absorbance from 190 to 600 nm, and later the chromatograms were analyzed at 254, 280, 320, and 365 nm. The mass spectra were simultaneously acquired using the ESI in the negative and positive ionization modes with a capillary voltage of 4.0 kV, fragmentation voltage of 130 V, skimmer voltage of 65 V, the gas and sheath gas (nitrogen) flows of 12 L/min each, and their temperatures of 350 and 400 °C, respectively. The mass spectra were recorded in the negative ionization mode, within the range of 40–1000 *m/z*, similarly to the MS/MS spectra, which were collected at two collision energy values of 10 and 20 V. Two highest signals obtained in MS1 spectra were selected for further fragmentation and after obtaining 1 spectrum of a given mass, the signal was excluded for the next 0.3 min. The nebulization pressure was 35.0 psig. The injection volume was set at 20 µL of each standard and tea infusion.

**Figure 3:**
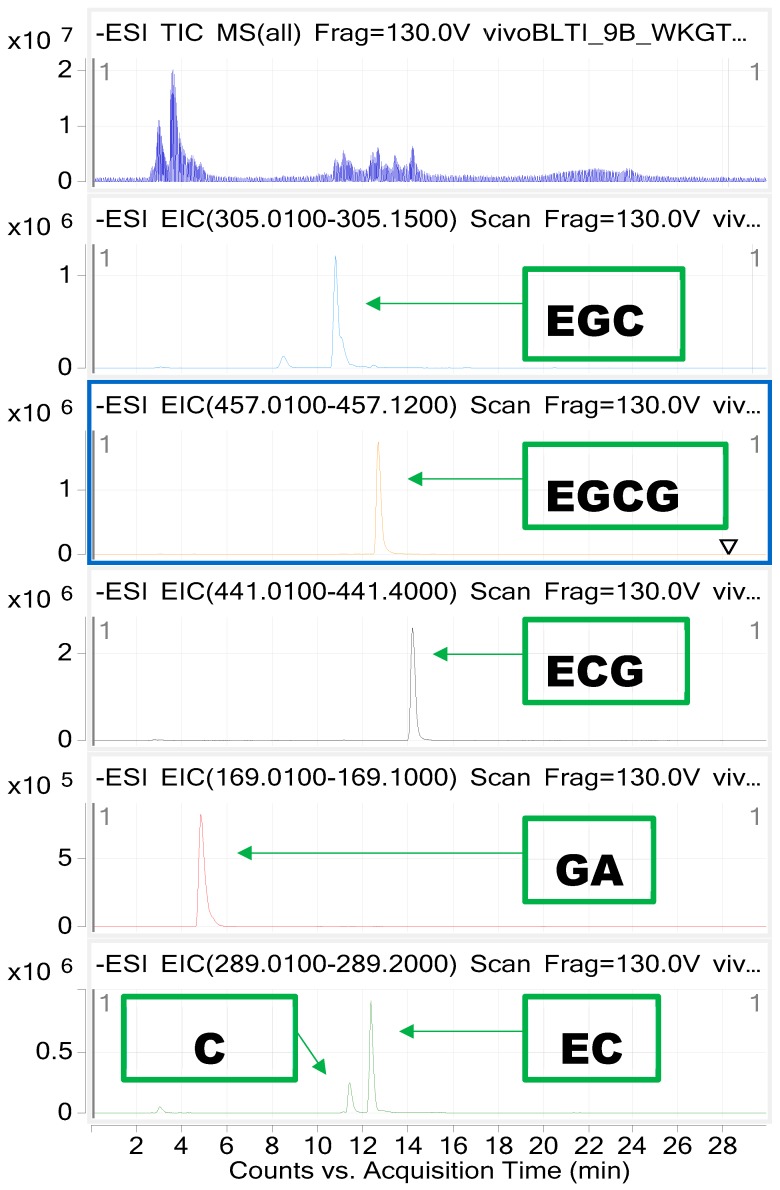
The EIC chromatograms of all catechins identified in the extracts in the negative mode of LC ESI-Q-TOF-MS analysis, identified based on the comparison with reference compounds.

Identification of each catechin was performed based on their retention times and mass spectra (see Figure 1 and Figure S1 in the Supplementary Material). The standards of all catechins and gallic acid (GA) were used in the concentration of 2 mg/mL (stock solution) and then several dilutions (to calculate a 5-point calibration curve) were performed to plot the calibration curves for each compound and characterized the method (calculation of limit of detection (LOD), limit of quantitation (LOQ) values and linearity of the method). The LOQ was determined as a triple value of the obtained LOD, which was calculated as 3 times the signal-to-noise ratio (S/N ratio) value. Results of quantitative analysis of all obtained extracts were evaluated based on the calibration curve equations for each catechin and GA. Table 4 presents the parameters of the method and Figure 3 presents the chromatograms.

### 3.5. Antioxidant Activity of Investigated Black Tea Infusions

#### 3.5.1. TPC

The analysis was conducted according to the modified method with Folin–Ciocalteu reagent, described elsewhere [34]. Briefly, 0.5 mL of each tea infusion was mixed with 30 mL of distilled water and 2.5 mL of Folin–Ciocalteu reagent. After 1 min (but no longer than 8 min), 7.5 mL of 20% Na_2_CO_3_ solution was added and the mixture was filled with distilled water to a total volume of 50 mL. After 2 h, the absorbance was measured at a wavelength of 760 nm in 1-cm cuvettes using a UV-Vis spectrophotometer (Thermo Fisher Scientific Evolution, Waltham, MA, USA). The blank was prepared according to the same protocol, but, instead of a sample, 0.5 mL of distilled water was added to the reaction mixture. Moreover to avoid sample′s inherent absorbance the corrections were made (absorbance of the solution of the sample, without Folin–Ciocalteu reagent). A calibration curve was performed with an aqueous solution of GA (50–500 mg/mL). For this purpose, instead of the analyte, 0.5 mL of aqueous solutions of GA was added at concentrations of 50, 100, 200, 300, and 500 mg/L. TPC in the studied samples was expressed as mg gallic acid equivalents (GAE) per 100 mL of tea infusions.

#### 3.5.2. DPPH Test

DPPH test was performed according to a previously described procedure with minor modifications [35]. Black tea infusion (0.2 mL each) was mixed with 3.8 mL of DPPH solution (6 × 10^−5^ M in methanol). The absorbance at 515 nm was read at t = 0 (AC_0_) and in 5-min intervals until the reaction reached the *plateu* value (AC_t_). For all samples, it was no longer than 30 min. A control sample was prepared by replacing the addition of extract with methanol. The obtained results were expressed as a percentage of inhibition using the following equation: inhibition [%] = [(AC_0_ − AC_t_)/AC_0_] × 100. To express the antioxidant potential of the investigated samples water solutions of Trolox in the concentration range 0–15 µM/L were prepared and used according to the same protocol. 

#### 3.5.3. Statistical Analysis

The statistical evaluation of data, including the principal component analysis (PCA), one-way Anova and post hoc tests, was performed in Statistica 12 program (StatSoft, Tulsa, OR, USA). The statistical significance of all obtained results was determined at *p* < 0.05.

## 4. Conclusions

This study shows a detailed analysis of catechins content, TPC analysis, and radical scavenging activity assessment of black tea samples from different cultivation areas: China, Japan, Kenya, Iran, Nepal, India, and Sri Lanka. EGC was the dominant catechin in the studied samples, followed by EGCG. The obtained results recorded by LC-ESI-Q-TOF-MS indicated the highest catechin content in black tea samples from India because of the presence of highest EGC concentration. These products were also characterized by the highest TPC and antiradical activity. However, the tea extracts from Iran were found to be the weakest source of catechins and the mildest antioxidant agent. 

PCA revealed that the EC, ECG, EGC, and EGCG content was not correlated with DPPH, GA, and TPC content; however, a strong correlation of EC and ECG between themselves was noted and a negative correlation of these two catechins and EGCG and EGC was revealed. Despite the above findings, the chemometric analysis revealed that the level of simple catechins in the black tea infusions still did not influence the antioxidant potential of the samples. In case of black teas, the antioxidant properties must be strongly influenced by other compounds of phenolic nature, possibly the condensed phenolics, which occur in the fermentation process of *C. sinensis* leaves [13].

Finally, the chemometric approach did not reveal any correlation between the black tea composition and the place of origin. However, the influence of the altitude of the cultivar on the tea quality was observed in case of tea samples from Kenya and Nepal, whose composition and antioxidant potential were very similar.

## Figures and Tables

**Table 1:** The content of gallic acid and catechins in the black teas.

Parameter	GA *	EGC *	EGCG *	ECG *	EC *	Total
**CH (China)**						
Mean (mg/100 mL)	3.54	18.0	0.29	-	-	**21.8**
SD	0.28	1.92	0.02			
Range	3.37–4.11	15.7–20.6	0.18–0.45			
**JA (Japan)**						
Mean (mg/100 mL)	2.66	14.2	0.15	-	0.04	**17.1**
SD	0.43	1.77	0.04		0.01	
Range	2.05–3.09	12.0–17.3	0.12–0.20		0.03–0.07	
**K (Kenya)**						
Mean (mg/100 mL)	5.34	33.0	0.12	-	0.38	**38.8**
SD	0.45	2.93	0.03		0.03	
Range	4.69–5.94	29.9–36.5	0.08–0.15		0.35–0.41	
**I (India)**						
Mean (mg/100 mL)	6.0	178.0	0.86	-	-	**184.8**
SD	0.73	19.5	0.07			
Range	5.28–7.11	156.8–205.2	0.64–0.96			
**S (Sri Lanka)**						
Mean (mg/100 mL)	3.74	49.2	1.18	-	-	**54.1**
SD	0.59	5.25	0.17			
Range	3.29–4.53	41.2–58.4	0.84–1.32			
**IR (Iran)**						
Mean (mg/100 mL)	0.27	9.91	-	0.08	0.28	**10.5**
SD	0.01	1.16		0.007	0.05	
Range	0.21–0.29	8.35–11.4		0.06–0.09	0.19–0.34	
**NH (Nepal)**						
Mean (mg/100 mL)	3.68	25.5	0.05	0.36	0.51	**30.1**
SD	0.35	4.18	0.009	0.04	0.08	
Range	3.18–3.95	18.5–32.8	0.04–0.06	0.33–0.43	0.42–0.57	
**NM (Nepal)**						
Mean (mg/100 mL)	9.42	33.9	0.06	0.12	0.57	**44.1**
SD	1.74	4.01	0.02	0.022	0.08	
Range	7.12–11.5	28.1–39.4	0.04–0.09	0.1–0.15	0.48–0.68	

* GA (gallic acid), EC (epicatechin), ECG (epicatechin gallate), EGC (epigallocatechin), and EGCG (epigallocatechin-3-gallate).

**Table 2:** Total phenolic content (TPC) and antiradical activity of selected black teas.

Tea	TPC * (mg/100 mL)	SD	Antiradical Activity (%)	SD	Trolox Equivalent (µM/L)	SD
CH (China)	17.5 ^a^	1.73	33.6	3.19	1.74	0.18
JA (Japan)	28.4 ^b^	2.62	35.6	5.28	1.85	0.16
K (Kenya)	49.9 ^c^	5.12	75.6	8.80	2.55	0.22
I (India)	58.2 ^c^	6.16	84.5	7.31	3.11	0.34
S (Sri Lanka)	33.8 ^b^	4.28	54.8	7.70	1.97	0.21
IR (Iran)	20.2 ^a^	3.11	31.8	4.20	1.51	0.14
NH (Nepal)	52.7 ^c^	4.78	82.6	9.43	3.00	0.22
NM (Nepal)	44.0 ^c^	5.05	73.4	8.48	2.38	0.23

* expressed as gallic acid equivalents; different letters by column are statistically significantly different at *p* < 0.05.

**Table 3:** The geographical origin and number of samples for the investigated black teas.

Code	Sampling Region	Number (Representatives)
CH	China	6
JA	Japan	6
K	Kenya	6
IR	Iran	6
NH	Nepal (border of the Darjeeling region, altitude >2000 m)	6
NM	Nepal (altitude 1500–1700 m)	6
I	India (Assam region)	6
S	Sri Lanka	6

**Table 4:** Validation parameters obtained in the optimized LC-MS method (*n* = 5).

Compound	LOD (ng/mL)	LOQ (ng/mL)	R^2^	Calibration Curve Equation	Linearity Range (µg/mL)
GA	0.52	1.56	0.9990	*y* = 2.4 × 10^7^*x* − 1.2 × 10^6^	0.030–30
EGCG	0.46	1.38	0.9988	*y* = 4.6 × 10^7^*x* − 2.8 × 10^6^	0.015–35
EGC	0.42	1.26	0.9987	*y* = 6.0 × 10^7^*x* − 1.9 × 10^6^	0.020–40
ECG	0.42	1.26	0.9992	*y* = 6.6 × 10^7^*x* − 1.2 × 10^6^	0.015–40
EC	0.42	1.26	0.9994	*y* = 7.8 × 10^8^*x* + 3.3 × 10^5^	0.010–40

## Supplementary Materials

Supplementary materials are available online.

## References

[1] Mejia E.G., Ramirez-Mares M.V., Puangpraphant S. (2009). Bioactive components of tea: Cancer, inflammation and behavior. Brain Behav. Immun..

[2] Marcos A., Fischer A., Rea G., Hill S.J. (1998). Preliminary study using trace element concentrations and a chemometrics approach to determine geographical origin of tea. J. Anal. Atom. Spectrom..

[3] Barua A. (2008). Romancing the Camellia assamica (Assam and the story of tea). Assam Rev. Tea News..

[4] Almajano P., Carbo R., Lopez Jimenez J.A., Gordon M.H. (2008). Antioxidant and antimicrobial activities of tea infusions. Food Chem..

[5] Zhang H.F., Shi Y.P. (2012). Magnetic retrieval of chitosan: Extraction of bioactive constituents from green tea beverage samples. Analyst.

[6] Yi T., Zhu L., Peng W.-L., He X.-C., Chen H.-L., Li J., Yu T., Liang Z.-T., Zhao Z.-Z., Chen H.-B. (2015). Comparison of ten major constituents in seven types of processed tea using HPLC-DAD-MS followed by principal component and hierarchical cluster analysis. LWT-Food Sci. Technol..

[7] Chacko S.M., Thambi P.T., Kuttan R., Nishigaki I. (2010). Beneficial effects of green tea: A literature review. Chin. Med..

[8] Hilal Y., Engelhardt U. (2007). Characterisation of white tea—Comparison to green and black tea. J. Verbe. Lebensm..

[9] Cantatore A., Randall S.D., Traum D., Adams S.D. (2013). Effect of black tea extract on herpes simplex virus-1 infection of cultured cells. BMC Complement. Altern. Med..

[10] Pan M.-H., Lai C.-S., Wang H., Lo C.-Y., Ho C.-T., Li S. (2013). Black tea in chemo-prevention of cancer and other human diseases. Food Sci. Hum. Wellness.

[11] Pauli E.D., Scarminio I.S., Tauler R. (2016). Analytical investigation of secondary metabolites extracted from *Camellia sinensis* L. leaves using a HPLC-DAD-ESI/MS data fusion strategy and chemometric methods. J. Chemom..

[12] Yao L.H., Jiang Y.M., Caffin N., D′Arcy B., Datta N., Liu X., Singanusong R., Xu Y. (2006). Phenolic compounds in tea from Australian supermarkets. Food Chem..

[13] Halder J., Bhaduri A.N. (1998). Protective role of black tea against oxidative damage of human red blood cells. Biochem. Biophys. Res. Commun..

[14] Lung H.L., Ip W.Q., Chen Z.Y., Mak N.K., Leung K.N. (2004). Comparative study of the growth-inhibitory and apoptosis-inducing activities of black tea theaflavins and green tea catechin on murine myeloid leukemia cells. Int. J. Mol. Med..

[15] Yang C.S., Wang X., Lu G.L., Picinisch S.C. (2009). Cancer prevention by tea: Animal studies, molecular mechanisms and human relevance. Nat. Rev. Cancer.

[16] Ferrazzano G.F., Amatoa I., Ingenitoa A., De Natale A., Pollio A. (2009). Anti-cariogenic effects of polyphenols from plant stimulant beverages (cocoa, coffee, tea). Fitoterapia.

[17] Friedman M. (2007). Overview of antibacterial, antitoxin, antiviral, and antifungal activities of tea flavonoids and teas. Mol. Nutr. Food Res..

[18] Bahorun T., Luximon-Ramma A., Neergheen-Bhujun V.S., Gunness T.K., Googoolye K., Auger C., Crozier A., Aruoma O.I. (2012). The effect of black tea on risk factors of cardiovascular disease in a normal population. Am. J. Prev. Med..

[19] Grassi D., Draijer R., Desideri G., Mulder T., Ferri C. (2015). Black tea lowers blood pressure and wave reflections in fasted and postprandial conditions in hypertensive patients: A randomised study. Nutrients.

[20] Masoum S., Heshamt S. (2015). Photoluminescence quantitative analysis of gallic acid and caffeine in green tea using multi-way chemometric approaches. Iran. J. Math. Chem..

[21] Khokhar S., Venema D., Hollmann P.C.H., Dekker M., Jongen W. (1997). A RP-HPLC method for the determination of tea catechins. Cancer Lett..

[22] Horie H., Kohata K. (1998). Application of capillary electrophoresis to tea quality estimation. J. Chromatogr. A.

[23] Wang H., Provan G.J., Helliwell K. (2000). Tea flavonoids: Their functions, utilisation and analysis. Trends Food Sci. Technol..

[24] Tao W., Zhou Z., Zhao B., Wei T. (2016). Simultaneous determination of eight catechins and four theaflavins in green, black and oolong tea using new HPLC–MS–MS method. J. Pharm. Biomed. Anal..

[25] Koch W., Kukula-Koch W., Głowniak K. (2017). Catechin composition and antioxidant activity of black teas in relation to brewing time. J. AOAC Int..

[26] Wiseman S.A., Balentine D.A., Frei B. (1997). Antioxidants in tea. Crit. Rev. Food Sci. Nutr..

[27] Han C., Chen J. The screening of active anticarcinogenic ingredients’ in tea. Proceedings of the 95 International Tea-Quality-Human Health Symposium.

[28] Fraser K., Harrison S.J., Lane G.A., Otter D.E., Hemar Y., Quek S.-Y., Rasmussen S. (2012). Non-targeted analysis of tea by hydrophilic interaction liquid chromatography and high-resolution mass spectrometry. Food Chem..

[29] Fraser K., Lane G.A., Otter D.E., Hemar Y., Quek S.-Y., Harrison S.J., Rasmussen S. (2013). Analysis of metabolic markers of tea origin by UHPLC and high-resolution mass spectrometry. Food Res. Int..

[30] Chen G.H., Yang C.Y., Lee S.J., Wu C.C., Tzen J.T.C. (2014). Catechin content and the degree of its galloylation in oolong tea are inversely correlated with cultivation altitude. J. Food Drug Anal..

[31] Han W.Y., Huang J.G., Li X., Li Z.X., Ahhamed G.J., Yan P., Stepp J.R. (2017). Altitudinal effects on the quality of green tea in east China: A climate change perspective. Eur. Food Res. Technol..

[32] Wang H., Helliwell K. (2001). Determination of flavonols in green and black tea leaves and green tea infusions by high-performance liquid chromatography. Food Res. Int..

[33] Su Y.L., Leung L.K., Huang Y., Chen Z.Y. (2003). Stability of tea theaflavins and catechins. Food Chem..

[34] Singleton V.L., Orthofer R., Lamuela-Raventos R.M. (1999). Analysis of total phenols and other oxidation substrates and antioxidant by means of Folin–Ciocalteu reagent. Methods Enzymol..

[35] Koch W., Baj T., Kukula-Koch W., Marzec Z. (2015). Dietary intake of specific phenolic compounds and their effect on the antioxidant activity of daily food rations. Open Chem..

