# Correction: Koch, W., et al. Black Tea Samples Origin Discrimination Using Analytical Investigations of Secondary Metabolites, Antiradical Scavenging Activity and Chemometric Approach. *Molecules* 2018, *23*, 513

**DOI:** 10.3390/molecules23092093

**Published:** 2018-08-21

**Authors:** Wojciech Koch, Wirginia Kukula-Koch, Łukasz Komsta

**Affiliations:** 1 Chair and Department of Food and Nutrition, Medical University of Lublin, 4a Chodźki Str., 20-093 Lublin, Poland; kochw@interia.pl; 2 Chair and Department of Pharmacognosy with Medicinal Plant Unit, Medical University of Lublin, 1 Chodźki Str., 20-093 Lublin, Poland; 3 Department of Medicinal Chemistry, Medical University of Lublin, 4 Jaczewskiego str., 20-090 Lublin, Poland; lukasz.komsta@umlub.pl

The authors wish to make the following corrections to their paper [1].

1. We found that in Affiliation section, in the affiliation No 2, it should be medicinal instead of medical. The correct affiliation No 2 is as follows: Chair and Department of Pharmacognosy with Medicinal Plant Unit, Medical University of Lublin, 1 Chodźki Str., 20-093 Lublin, Poland

2. We found that in Table 2, it should be mM instead of µM. The correct Table 2 is as follows:

3. We found that in Section 3.5.2., there are three errors that we made during the revision process. The corrected Section 3.5.2. is as follows:

3.5.2. DPPH Test

DPPH test was performed according to a previously described procedure with minor modifications [35]. Black tea infusion (0.1 mL each) was mixed with 3.9 mL of DPPH solution (6 × 10^−5^ M in methanol). The absorbance at 515 nm was read at *t* = 0 (AC_0_) and in 5-min intervals until the reaction reached the *plateu* value (AC_t_). For all samples, it was no longer than 30 min. A control sample was prepared by replacing the addition of extract with methanol. The obtained results were expressed as a percentage of inhibition using the following equation: inhibition [%] = [(AC_0_ − AC_t_)/AC_0_] × 100. To express the antioxidant potential of the investigated samples, water solutions of Trolox in the concentration range 0–15 mM/L were prepared and used according to the same protocol.

The authors would like to apologize for any inconvenience caused to the readers by these changes which do not affect the scientific results. The manuscript will be updated and the original will remain on the article webpage, with a reference to this Correction.

## Figures and Tables

**Table 2:** Total phenolic content (TPC) and antiradical activity of selected black teas.

Tea	TPC * (mg/100 mL)	SD	Antiradical Activity (%)	SD	Trolox Equivalent (mM/L)	SD
CH (China)	17.5 ^a^	1.73	33.6	3.19	1.74	0.18
JA (Japan)	28.4 ^b^	2.62	35.6	5.28	1.85	0.16
K (Kenya)	49.9 ^c^	5.12	75.6	8.80	2.55	0.22
I (India)	58.2 ^c^	6.16	84.5	7.31	3.11	0.34
S (Sri Lanka)	33.8 ^b^	4.28	54.8	7.70	1.97	0.21
IR (Iran)	20.2 ^a^	3.11	31.8	4.20	1.51	0.14
NH (Nepal)	52.7 ^c^	4.78	82.6	9.43	3.00	0.22
NM (Nepal)	44.0 ^c^	5.05	73.4	8.48	2.38	0.23

* expressed as gallic acid equivalents; different letters by column are statistically significantly different at *p* < 0.05.
