# Supplementary material for: Black Tea Samples Origin Discrimination Using Analytical Investigations of Secondary Metabolites, Antiradical Scavenging Activity and Chemometric Approach
Source: Molecules. 2018 Feb 26;23(3):513. doi: 10.3390/molecules23030513 (PMC6017602; doi:10.3390/molecules23030513)
Supplement: Supplementary file 1 [file molecules-23-00513-s001.pdf]

SUPPLMENTARY MATERIAL

Black tea samples origin discrimination using analytical investigations of secondary metabolites, antiradical scavenging activity and chemometric approach

Wojciech Koch, Wirginia Kukula-Koch, Łukasz Komsta

Figure S1. Fragmentation patterns of the investigated catechins and gallic acid recorded in the negative ionization mode of LC-ESI-Q-TOF-MS spectrometry (the Y axis values for more clear view are divided by 10).

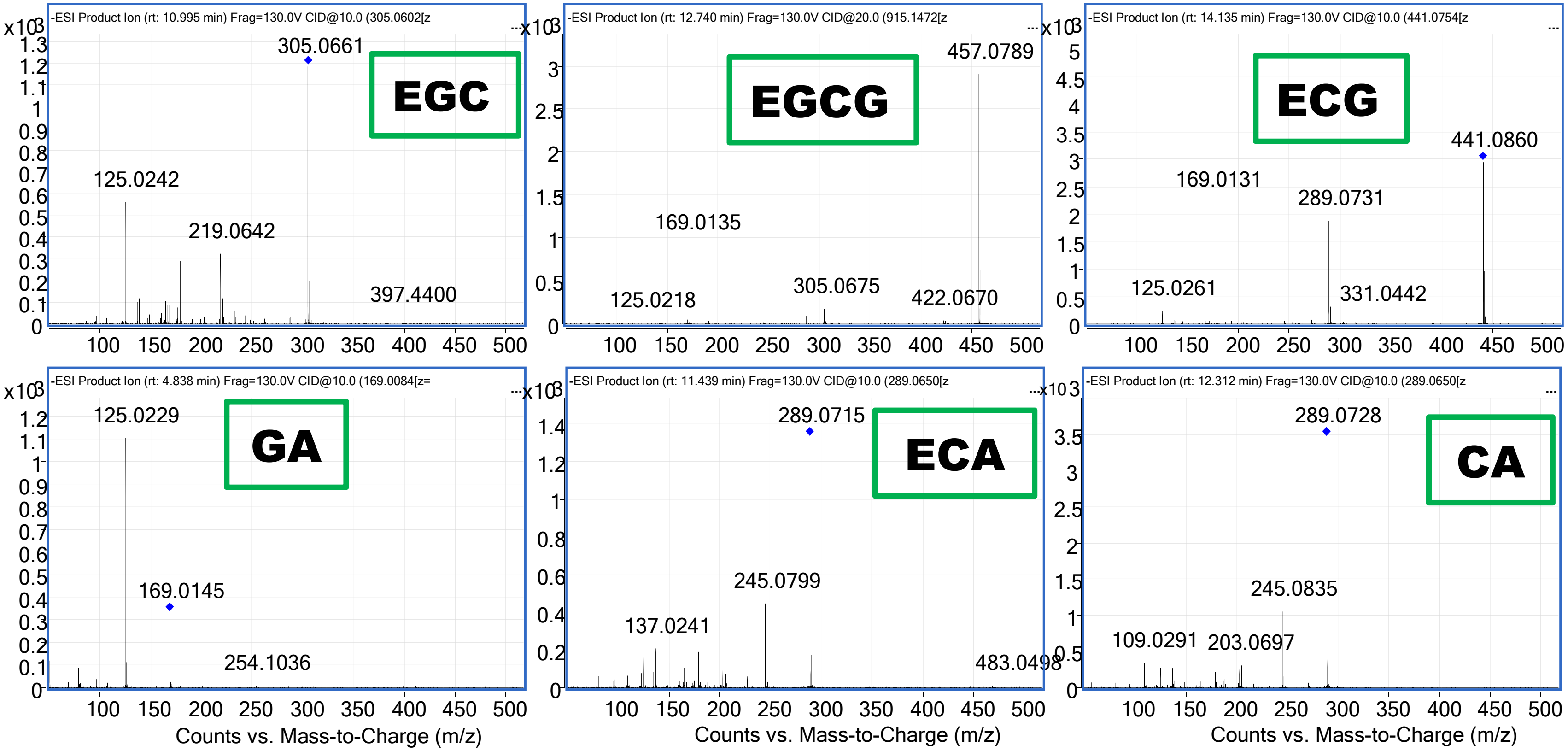

Figure S2. Statistical significance of each parameter, separately.

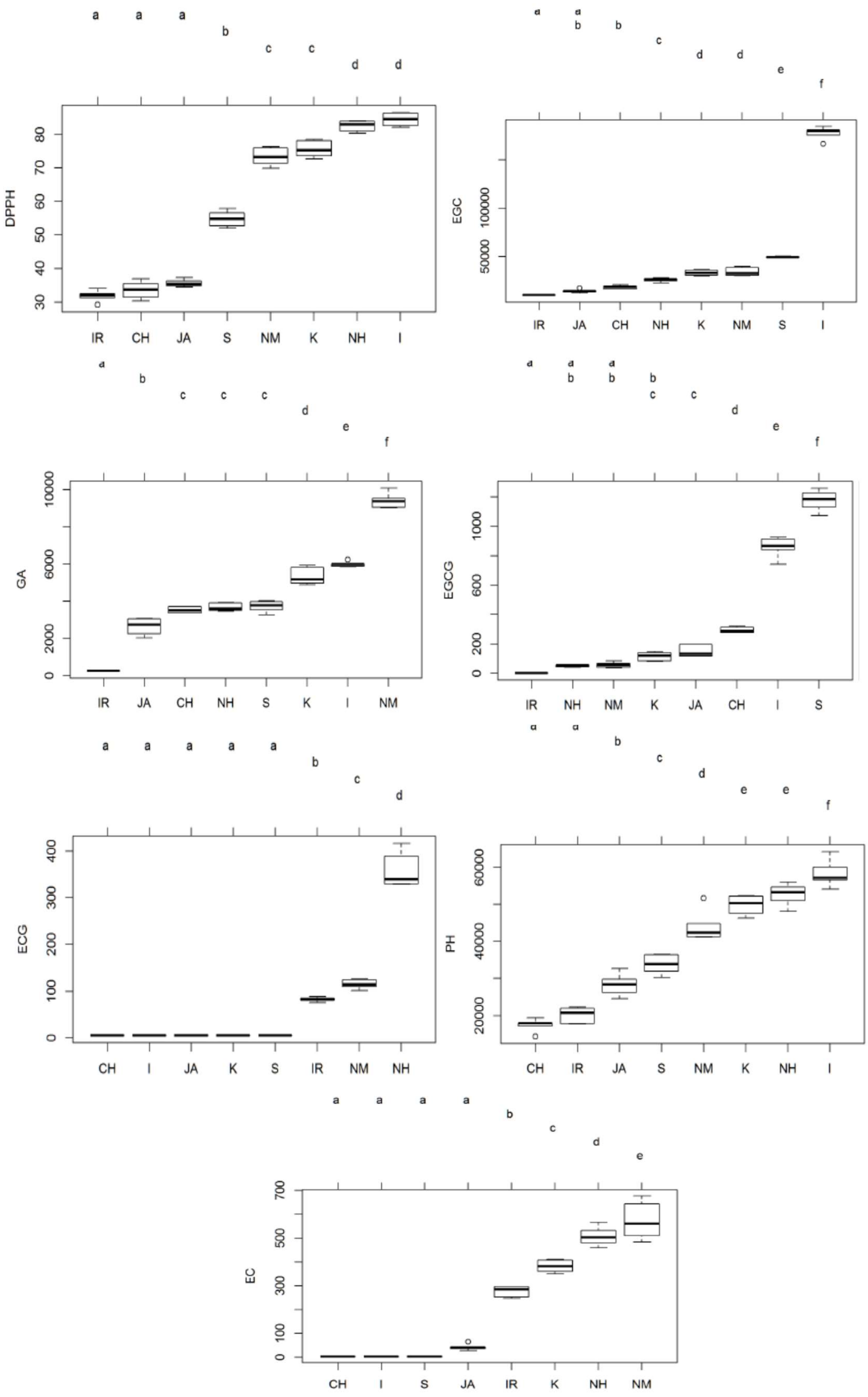

Table S1. Correlation of individual parameters in pairs and its significance.

	lower	r	upper	p
**DPPH-GA**	0.4716	0.6664	0.7992	2.334e-07
**DPPH-EGC**	0.3231	0.5562	0.7256	4.052e-05
**DPPH-EGCG**	-0.1464	0.1437	0.411	0.3299
**DPPH-ECG**	0.1233	0.3937	0.6096	0.005637
**DPPH-EC**	0.204	0.4614	0.6591	0.0009635
**DPPH-PH**	0.92	0.9546	0.9744	0
**GA-EGC**	0.0888	0.3638	0.5872	0.01104
**GA-EGCG**	-0.2137	0.07496	0.3516	0.6126
**GA-ECG**	-0.2788	0.005824	0.2895	0.9687
**GA-EC**	0.1149	0.3864	0.6042	0.00667
**GA-PH**	0.3686	0.5909	0.7492	9.802e-06
**EGC-EGCG**	0.3943	0.6101	0.7621	4.157e-06
**EGC-ECG**	-0.481	-0.228	0.05998	0.119
**EGC-EC**	-0.5667	-0.3368	-0.05828	0.01923
**EGC-PH**	0.3855	0.6035	0.7577	5.607e-06
**EGCG-ECG**	-0.6251	-0.4147	-0.148	0.003385
**EGCG-EC**	-0.7894	-0.6513	-0.4506	5.35e-07
**EGCG-PH**	-0.1401	0.15	0.4164	0.3087
**ECG-EC**	0.4796	0.6721	0.8029	1.684e-07
**ECG-PH**	0.05044	0.3298	0.5614	0.02205
**EC-PH**	0.1034	0.3765	0.5968	0.008348

Table S2. The raw data obtained from Principal Components Analysis.

Principal Components Analysis

	PC1	PC2	PC3	PC4	PC5	PC6	PC7
**DPPH**	0.5504	-0.04181	0.1044	-0.001019	0.2546	-0.06312	0.7846
**GA**	0.4204	-0.06352	-0.6959	-0.3386	-0.4034	-0.2212	-0.09303
**EGC**	0.2985	-0.4795	0.1619	0.4605	-0.5027	0.432	-0.05806
**EGCG**	0.03593	-0.5603	0.2326	-0.7094	0.2131	0.2521	-0.1358
**ECG**	0.2375	0.4339	0.6101	-0.3221	-0.4986	-0.1582	-0.07601
**EC**	0.3013	0.5057	-0.1662	-0.07693	0.2237	0.7341	-0.1759
**PH**	0.5317	-0.07977	0.1572	0.2456	0.4192	-0.3647	-0.5632

	PC1	PC2	PC3	PC4	PC5	PC6	PC7
**Standard deviation**	1.779	1.572	0.8626	0.5737	0.4149	0.3002	0.1679
**Proportion of Variance**	0.4521	0.3531	0.1063	0.04702	0.02459	0.01288	0.00403
**Cumulative Proportion**	0.4521	0.8052	0.9115	0.9585	0.9831	0.996	1

Table S3. The analysis of variance (ANOVA) for all parameters.

Parameter	F value	Pr(>F)
**DPPH**	731.7	3.969e-40
**GA**	489.9	1.112e-36
**EGC**	1936	1.566e-48
**EGCG**	732.6	3.876e-40
**ECG**	492.7	9.932e-37
**EC**	332.7	2.263e-33
**PH**	176.9	5.002e-28

Figure S3. Biplot figure.

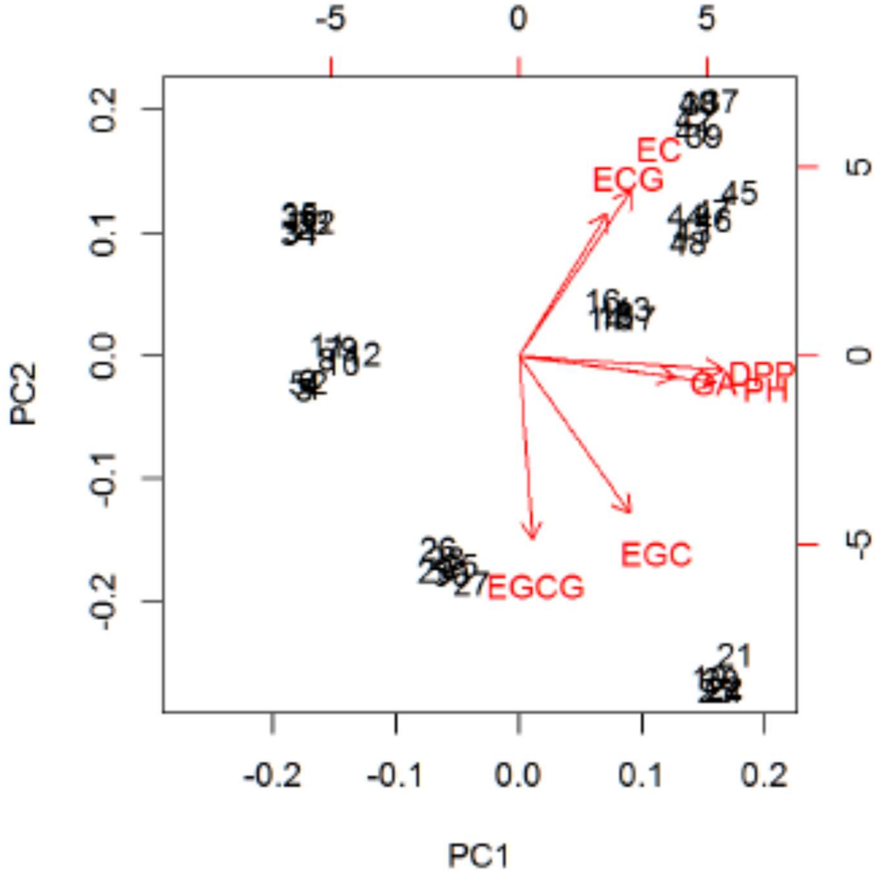

Table S4. The analysis of variance (ANOVA) for each parameter separately.

	Df	Sum Sq	Mean Sq	F value	Pr(>F)
**DPPH**	7	21835	3119	731.7	3.969e-40
**DPPH**	40	170.5	4.263	NA	NA
**GA**	7	302171663	43167380	489.9	1.112e-36
**GA**	40	3524580	88115	NA	NA
**EGC**	7	1.275e+11	1.822e+10	1936	1.566e-48
**EGC**	40	376468542	9411714	NA	NA
**EGCG**	7	7977818	1139688	732.6	3.876e-40
**EGCG**	40	62230	1556	NA	NA
**ECG**	7	634588	90655	492.7	9.932e-37
**ECG**	40	7360	184	NA	NA
**EC**	7	2449540	349934	332.7	2.263e-33
**EC**	40	42075	1052	NA	NA
**PH**	7	9.891e+09	1.413e+09	176.9	5.002e-28
**PH**	40	319581667	7989542	NA	NA
